# Motivational orientation and perception of active learning instruction by pre-service language teachers

**DOI:** 10.3389/fpsyg.2024.1307733

**Published:** 2024-12-23

**Authors:** Florentina Halimi, Marta Tryzna, Angela Brunstein

**Affiliations:** ^1^College of Arts and Science, The Gulf University for Science and Technology, Mishref, Kuwait; ^2^School of Arts and Science, American International University, Al Jahra, Kuwait

**Keywords:** motivaion, achievement goal orientation, active instruction, engagement, preservice language teachers

## Abstract

This study investigates the impact of active learning instruction on the motivational orientation of pre-service language teachers. The data were collected by using the AGQ-R and the StRIP questionnaire, and analyzed through repeated measures of MANOVAs and correlation coefficient. Pre-service language teachers reported a higher approach goal orientation emphasizing the desire to succeed rather than avoidance goal orientation, i.e., the fear of failure. In terms of classroom practices, the participants report having experienced all types of instruction (passive, active, interactive and constructive) in the language education program. Although the pre-service teachers’ perception of instruction as passive is preponderant in this study, it does not necessarily point to a higher use of traditional passive lecture and note taking practices. Regardless of the perceived instruction type, the participants’ motivation and engagement remain high, as they report high cognitive and emotional engagement, as well as very high participation and low distraction levels. Regarding correlations, motivation, engagement and active instruction are all highly correlated with each other, as highly motivated and engaged individuals tend to benefit more from active instruction than less motivated students, who chiefly rely on passive instruction for academic success. The results of the study may indicate an emergent need for a careful balance between various teaching strategies and approaches in language education programs in particular, and possibly at tertiary level pedagogy in general.

## 1 Introduction

Pre-service language teachers, who will lead their classrooms one day, hold a significant responsibility to transform the educational environment, to provide their students with valuable tools for success and to help their students achieve success and promote well-being in the classroom (Daniels et al., 2017; Dao et al., 2018; Tuytens and Devos, 2018; Nichols and Brewington, 2020). Motivation is a complex concept that significantly impacts pre-service teachers’ thoughts, emotions, and actions, ultimately playing a vital role in determining their students’ eagerness to learn and accomplish achievement (Fray and Gore, 2018; Tang et al., 2020). Understanding pre-service teachers’ motivational approach is important for creating an encouraging learning environment and enhancing their educational skills (Stevenson et al., 2017; Brandt et al., 2021). Active instruction in the education program has been found to be a successful kind of interaction between education faculty and pre-service teachers and has direct implications for their teacher training journey (Cirillo et al., 2022; Candel et al., 2024). The move toward active instruction in education programs is not solely driven by theory and research but also by new educational requirements.

Active instruction provides a great opportunity to enhance learning and promote meaningful experiences for pre-service teachers to develop their teaching philosophy based on contemporary educational requirements. While several studies have examined active instruction with pre-service teachers (O’Grady et al., 2014; Dağ et al., 2019; Caruso, 2021; Dearamae et al., 2021; Näykki et al., 2021; Candel et al., 2024), there are very few that examined their motivation related to active instruction in classrooms.

Building on the foundation laid by previous research on active learning, this study was conducted in Kuwait, a developing nation in the Middle East with a growing emphasis on educational priority. A brief description of the education system in Kuwait can shed light on the dire need for implementing active instruction strategies by English language teachers in public schools. Public school teachers strictly follow the framework prescribed by the Ministry of Education in terms of the curriculum, instructional materials and assessment (Tryzna and Al Sharoufi, 2017). English is taught as a foreign language one hour a day from first to twelfth grade by teachers who share the students’ first language, Arabic (Alsafran et al., 2020; Alnwaiem et al., 2021) and who use it not just to manage classroom behavior but crucially to explain English grammar and vocabulary, often relying on the grammar-translation method (Alotaibi et al., 2014; Alenezi et al., 2021). Currently, English is not the medium of instruction in the English language classrooms in the public school system and the traditional teaching methods, which favor memorization, prevail (Alazemi and Alenezi, 2022).

The aim of this research was to investigate the impact of the types of instructions used by the faculty and students’ perceptions on achievement motivation in a private American university in Kuwait. This paper is part of the research project that was initiated after a careful analysis of the institutional curriculum and the course syllabi, which incorporate active learning objectives and focus on critical thinking, group work, and hands-on activities. Specifically, the study focused only on students majoring in language education since they are trained to use active teaching methods in their future classrooms. By selecting pre-service teachers, the study aimed to gain better insights into their views on active instruction from both learning and teaching perspectives.

The results of this study show that pre-service teachers’ perception of both active and passive instructions utilized in the language education program was positive. This may indicate the need for a sense of balance between various teaching strategies and approaches in language education programs, and possibly at tertiary level pedagogy in general. As students’ motivation and engagement levels necessarily vary within and across university programs, balancing passive and active instruction and varying educational approaches might ensure better knowledge and skills acquisition, as well as laying the foundations for future academic success.

## 2 Review of literature

### 2.1 Academic motivation

Human motivation and behavior have been considered psychological frameworks in the Self-Determination Theory (Deci and Ryan, 1985), which distinguishes between different types of motivation, leading to optimal development, well-being, and change in behavior. Self-determination theory (STD) has been widely used in research, providing insights into student motivation and engagement. Based on STD, Ahmadi et al. (2023) emphasized that teacher behavior has a strong influence on student motivation in educational settings and offered a classification for teacher behaviors in accordance with STD concept.

Achievement motivation research has long been focused on student goal orientations in various educational environments (Elliot et al., 2011). This theoretical framework, originally developed by Elliot and Harackiewicz (1996), was initially tested with undergraduates in large lecture-based introductory courses (Harackiewicz et al., 2000, 2002) and later validated in small advanced-level seminars (Barron and Harackiewicz, 2003). It was subsequently revised and refined (Elliot and McGregor, 2001; Elliot, 2006; Elliot and Murayama, 2008) so that the construct’s best-known and most widely applied version is a 2×2 framework incorporating two types of achievement goals, i.e., mastery and performance, and two types of goal orientation, i.e., approach and avoidance orientations. The most recent version of the construct also includes a task element resulting in an expanded 3×2 framework (Elliot et al., 2011). However, the earlier 2×2 framework remains most widely utilized in the bulk of achievement-related studies with diverse populations across various educational contexts (Miller, 2022).

The 2×2 framework combines mastery and performance goals with approach and avoidance orientations. *Mastery* refers to competence acquisition in terms of intrapersonal standards, while *performance* is normative and relies on comparisons to the achievements of others. Mastery is rooted in an internal desire to acquire necessary skills, whereas performance is outward-oriented, whereby a sense of accomplishment is derived through positive reflection on peers (Phillips and Gully, 1997; VandeWalle et al., 2001). In terms of goal pursuit strategies, *approach* is a positive orientation toward a goal, i.e., a desire to succeed, while *avoidance* is a negative orientation, i.e., a desire not to fail. Taken together, four achievement goal orientations are produced forming the 2×2 matrix: mastery-approach (to acquire competence), mastery-avoidance (to avoid incompetence), performance-approach (to outperform others), and performance-avoidance (not to perform worse than others).

In teacher education research, pre-service teachers’ achievement goal orientation holds a significant position. It refers to their perceptions and goals pertaining to academic success, as well as their strategies and responses to learning difficulties (Castro-Villarreal et al., 2014). Obtaining a comprehensive understanding of pre-service teachers’ achievement goal orientation can provide crucial insights into their overall mindset, drive, and career growth (Butler, 2007, 2012; Smith et al., 2013).

Several studies (Elliot and McGregor, 2001; Smith et al., 2013) revealed that the majority of PSLTSs are motivated by their passion for learning and improving (mastery approach), while others were primarily driven by external rewards and recognition (performance-approach). The studies highlight numerous benefits that impact both teachers and students and promote knowledge and skills, resulting in successful academic outcomes (Butler, 2007, 2012).

Previous research indicates that achievement goal orientation of pre-service teachers has a significant impact on their teaching practices. According to a study conducted by Nguyen et al. (2021), future teachers who placed higher importance on mastering their skills showed significantly greater levels of effectiveness in teaching. In addition, they highlighted the importance of enhancing the quality of learning experiences to establish a classroom environment that is supportive and engaging. This emphasizes the key role that mastery approach orientation plays in developing effective teaching methods. On the other hand, PSLTs who prioritize performance goals are inclined toward achieving instructional efficiency and task completion rather than enhancing the quality of learning experiences.

**Figure 1:**
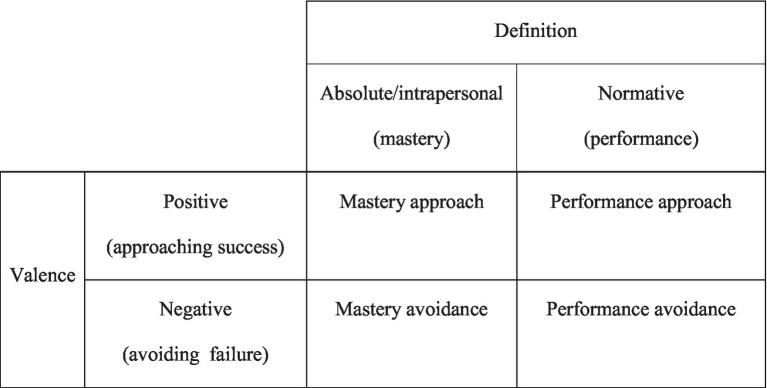
The 2×2 achievement goal framework (Elliot and McGregor, 2001, p. 502).

Another study (Wang et al., 2017) examined Canadian practicing teachers’ achievement goals, classroom goal structures, and teacher-related emotions. The results of the study have shown that teachers’ achievement goals play a significant role in predicting both classroom goal structures and teaching-related emotions. These effects are mediated by the influence that classroom goal structures have on the relationship between teachers’ goals and their emotional experiences during teaching (see Figure 1 for combination of concepts used in this study).

Essentially, the type of goals that teachers set for themselves can have a ripple effect on the classroom environment and their own emotional states, both of which are important factors for effective teaching and learning. Research studies on pre-service teachers’ achievement goal orientation (Butler, 2007, 2012; Smith et al., 2013; Castro-Villarreal et al., 2014; Wang et al., 2017; Nguyen et al., 2021) indicate a significant impact on their motivation, attitudes, and teaching practices. A mastery approach orientation may indicate a strong commitment to learning, self-improvement, and professional growth. However, a performance goal orientation may prioritize external validation over these important factors.

### 2.2 Forms of active instruction

In addition to the heightened focus on achievement goal orientation, the benefits of implementing active learning instruction in higher education have received increased attention among practitioners and researchers alike, in spite of the fact that this type of pedagogy has not been widely adopted by university instructors (Michael, 2007; Bovill et al., 2016). Active instruction relies on reconceptualizing the role of students from the traditional one, i.e., as passive knowledge recipients, typically ascribed to students in large lecture-based university classes, toward a more engaged one, where they are treated as co-creators of the classroom experience. Active instruction includes meaningful hands-on collaborative activities. Those help students to integrate new information and to internalize content through frequent multidirectional feedback, to focus on conceptual learning, and emphasizes on metacognitive awareness about the subject matter (Cavenagh, 2016).

Theobald et al. (2020) found in a meta-analysis of over 25 studies with over 50,000 university students enrolled in STEM classes that course passing rates can be improved by 33% while achievement gaps in the exam scores can be narrowed by 45% as a result of implementing high-quality active learning strategies in the classroom. The gains were especially significant in the case of students from non-traditional backgrounds (e.g., first-generation college enrollees), leading the researchers to conclude that active approaches to instruction have a potential to promote equity and excellence in higher education. Several studies have investigated the impact of active instruction on pre-service teachers’ knowledge acquisition, skill development, and attitudes toward teaching (O’Grady et al., 2014; Dağ et al., 2019; Caruso, 2021; Dearamae et al., 2021; Näykki et al., 2021; Candel et al., 2024). For instance, Candel et al. (2024) conducted a study on pre-service teachers’ perception of active learning in an online environment using flipped classrooms and gamification. The study results indicated that pre-service teachers appreciated the active learning strategies and their interest and motivation toward learning improved. In addition, active learning instruction has been associated with increased motivation, engagement, and self-efficacy among pre-service teachers (González-Gómez et al., 2022; Candel et al., 2024). Many studies have revealed that pre-service teachers with prior experience with active instruction were more inclined toward incorporating it in their future teaching practices (Valtonen et al., 2021). Providing pre-service teachers with training and support in using the different forms of active instruction is highly advantageous. One such approach is the professional development program emphasized in Näykki et al.’s (2021) study, which positively impacted pre-service teachers’ confidence and perception in implementing active instruction. The training can equip pre-service teachers with the essential knowledge and skills to effectively use active instruction in their classrooms. While numerous studies advocate active instruction and its effectiveness, several studies report that pre-service teachers may face difficulties using it, particularly when they are unfamiliar or uncomfortable with non-traditional teaching methods. Their concerns often revolve around classroom management and the constraints of time (Ellerton, 2013; O’Grady et al., 2014; Sahin-Taskin, 2018). O’Grady et al. (2014) examined pre-service and in-service science teachers’ perceptions of active instruction and its effectiveness in Irish second-level classrooms with a two-phase study. In the second phase, pre-service science teachers, assisted by teacher educators, designed a comparative test for students in lower secondary science classrooms, testing achievement differences between traditional and active learning approaches. While the test results show a significant difference between traditional and active learning for students’ performance, the majority of teachers were not convinced of its value. Since active instruction relies on high levels of student engagement, they have been found to promote not only cognitive benefits, such as deeper learning of the subject matter and enhanced critical thinking skills (Prince, 2004), well-focused attention and memory consolidation (Cavenagh, 2016), but also pedagogical gains such as better learning outcomes and lower failure rates (Freeman et al., 2014). In terms of positive emotional experiences in the classroom, active pedagogy leads to increased levels of motivation (Owens et al., 2017) and a heightened sense of achievement and personal development (Kuh et al., 2017). Given the multiple cognitive, pedagogical, and affective benefits of active instruction, it appears advantageous to link this pedagogical practice with the well-established psychological construct of achievement goal orientation in an effort to reexamine the various underpinnings of this construct and to test it in a novel context.

To analyze the effect of the instructional types and their relationship, the Student Response of Instructional Practices (StRIP, DeMonbrun et al., 2017) instrument has been used widely by researchers and practitioners. It measures the type of instruction ranging from traditional to various active instructional forms such as interactive, constructive, and active learning.

**Figure 2:**
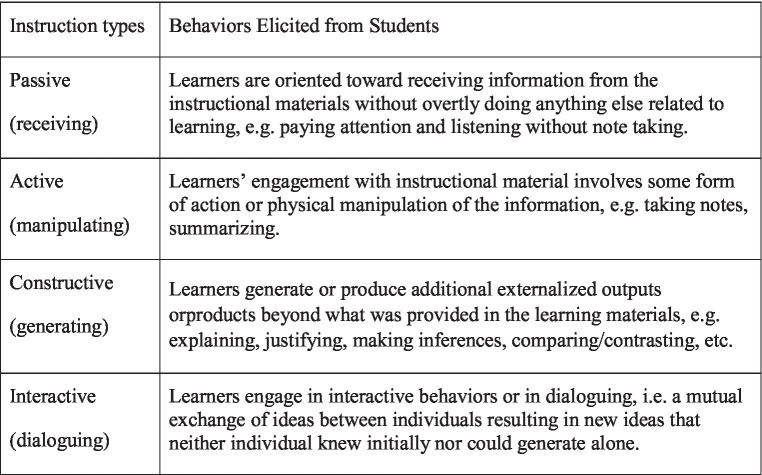
Four types of instruction (Chi and Wylie, 2014).

**Figure 3 fig3:**
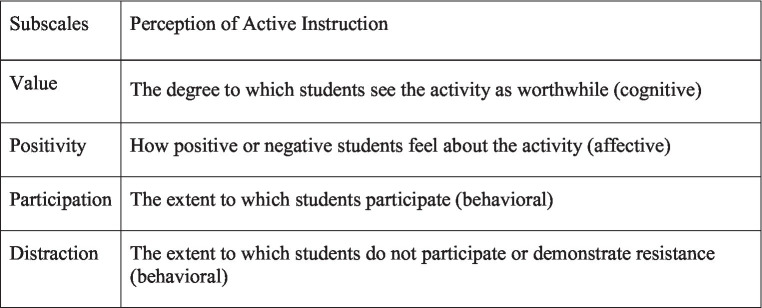
The subscales of student perceptions of classroom instruction (DeMonbrun et al., 2017).

**Figure 4:**
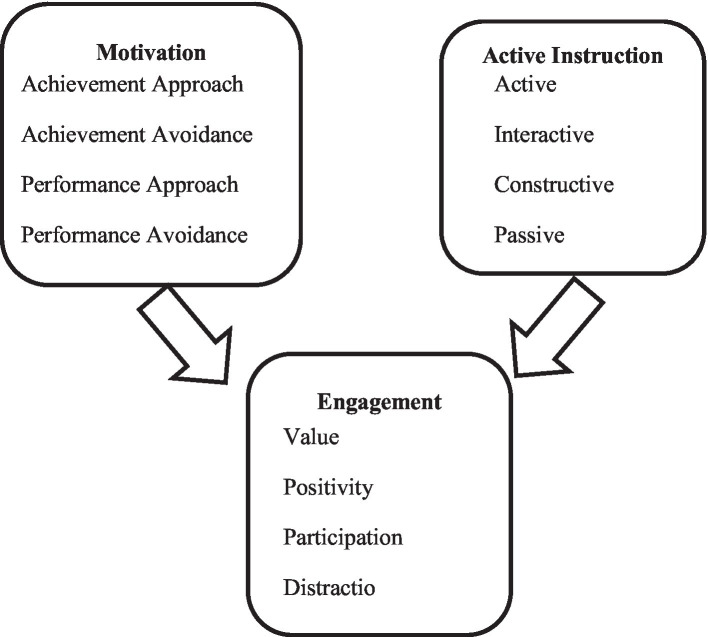
Versions of perceived instruction, motivation (i.e., achievement goal orientation), and engagement.

*Active instruction* was defined as a method used to engage students with activities by asking the instructor questions during class. *Interactive instruction* involves learning in groups by participating in hands-on activities during class, and students are graded based on their group performance. *Constructive* instruction involves self-directed learning and requires self-discovery rather than direct instruction. These instructional types have been reported to generate students’ resistance because it requires high expectations and differs significantly from traditional classes (Chi and Wylie, 2014; DeMonbrun et al., 2017) (see Figures 2 [Fig fig3]–4).

To analyze students’ perceptions of various types of instruction and address instructors’ concerns related to students’ resistance toward active instruction, the concept of classroom engagement was incorporated. Classroom engagement refers to how students respond to activities ranging from complete engagement to passive instances of boredom (Lawson and Lawson, 2013; DeMonbrun et al., 2017). Four forms of classroom engagement have been identified: (i) *cognitive engagement*, which refers to psychological investment in classroom activities; (ii) *affective-emotional engagement* pertaining to social and emotional connections to the classroom; (iii) *behavioral engagement* referring to students’ behavior in the classroom; and (iv) *instructor evaluation* referring to the students’ feedback on the instructor or course upon completion of the term. In addition, four variables measuring students’ perception of a type of instruction were used, such as cognitive value, emotional positivity, participation or resistance.

In spite of solid neurological and psychological underpinnings of various forms of active instruction, the stakeholders’ perceptions about such types of instruction and their purported benefits remain ambivalent. A study by Park et al. (2021) found that students in classrooms involving widely implemented active instruction have a somewhat negative perception regarding the practicality of activities and the extent of educational gains as compared to peers in traditional (i.e., lecture-based) classrooms. In other words, even though active learning strategies have been demonstrated to be effective pedagogical tools, students themselves tend to regard them as less useful for comprehending new information or internalizing knowledge.

The present study attempts to correlate active pedagogy with achievement goal orientations of students in the English Language Education program at a private English-medium university in Kuwait with an American curriculum. Language education is a particularly relevant context for implementing all forms of active instruction and investigating the development of this pedagogy in relation to achievement goal orientation, as the current students in the program are at the same time future language teachers in the public schools. Not only can the active learning approach benefit the current Education Program students in terms of their personal cognitive, educational, and emotional benefits, but it can also serve as a good model for creating positive classroom experiences in their future professional capacity as language teachers (Wang et al., 2021).

The rationale of the current study and questions are: the literature review has shown that active instruction (AI) and student engagement (SE) affect the student’s achievement goal orientations (AGO). The majority of research suggests that education students, pre-service and in-service teachers with mastery-approach orientation are highly motivated to succeed, and it is equally true that some educators with performance-approach orientation prioritize external validation. This study investigated the relationship between the constructs of 217 pre-service teachers’ perceived instruction, motivation, and engagement. More specifically, the following research questions were explored:

RQ 1: What is the motivational orientation of pre-service language teachers in terms of achievement goal orientation?

Previous research has indicated that pre-service teachers are highly motivated (Elliot and McGregor, 2001; Smith et al., 2013), most of them achievement approach oriented, but others performance approach oriented (Elliot and McGregor, 2001; Smith et al., 2013). This study aimed to replicate that result and quantify the proportions of different goal orientations within this pre-service teacher cohort. This is especially important because there is comparably little research using these tools for this student population.

RQ2: How do pre-service teachers’ perceptions vary across different types of instruction used in the classroom?

Most previous research compared the impact of active instruction to traditional teaching and has highlighted the benefits of active instruction (Theobald et al., 2020). At the same time, there is reported resistance against novel, unfamiliar ways of teaching by students (Park et al., 2021). Students who do not like and do not participate in active instruction are less likely to experience the reported benefits and less likely to later implement these methods in their own classes as a teacher. The current curriculum for pre-service teachers is active instruction based. Therefore, students are familiar with and used to active instruction. This study investigates their perception of different aspects of active instruction. Based on previous research (Kuh et al., 2017; Owens et al., 2017; González-Gómez et al., 2022; Candel et al., 2024), active instruction has been associated with increased motivation, engagement, and self-efficacy. Therefore, it was expected that the perception of active instruction is positively related to pre-service language teachers’ motivation as well as to engagement. This should be especially true for pre-service language teachers with achievement approach orientation. It is not clear yet, what the relationship might look like for pre-service language teachers with performance-approach instruction.

Despite the fact that some students raise objections against the active learning approach (Park et al., 2021), they may nevertheless benefit from active instruction (Theobald et al., 2020). Therefore, there seems to be a mismatch between the somewhat negative perception of active learning strategies by students, who may find them burdensome and effort-intensive (Walker et al., 2008; Minhas et al., 2012; Brazeal et al., 2016; Nguyen et al., 2021), and the actual effectiveness of such student-centered strategies in terms of cognitive benefits, i.e., improved comprehension and knowledge retention (Cavenagh, 2016), as well as emotional and behavioral gains, i.e., increased motivation and improved emotional health (Owens et al., 2017). In order to explore this apparent contradiction, the present study focuses on the relationship between learning strategies as perceived by student teachers, their motivation in terms of achievement goal orientation as well as cognitive, emotional, and behavioral aspects of engagement.

## 3 Method

### 3.1 The participants

In the set of 540 students from different universities in the region answering the AGQ-R (Elliot and McGregor, 2001) and a subset of questions from the StRIP (DeMonbrun et al., 2017), 137 were English education students at an American private university in Kuwait, who agreed to participate in this study. These students were reported to be 96 female and 41 males; 127 were local students, and 10 were international. On average, they were 23 years old, SD = 3.6 (range 18–38 years) and were, on average, in their 4th year of college (range 1–7). Their average GPA was 3.0 (SD = 0.56).

The English education students were selected only for the current study because the syllabi for the education courses emphasize the use of active instruction techniques, which require students to engage with the course material through a variety of methods, such as group discussions, problem-solving activities, and hands-on projects. Students must complete all required education courses (Levels 1–4) listed in the major sheet. According to academic regulations, the standard course load for education students is fifteen credit hours per semester or four to five courses, mostly with three credit hours. Students can take education courses covering various topics such as teaching and learning methods, classroom management, psychology, assessment, research, technology, and material development. The program also offers internships and teaching experience to help students gain practical experience.

Five professors are teaching the education courses, and guest experts are invited to supplement the education professors teaching education courses. All professors are expected to follow active instructions, creating an engaging and dynamic classroom environment that encourages students to learn and grow. The education professors undergo peer reviews to ensure these teaching methods are effective, providing valuable feedback to improve their teaching and course materials.

### 3.2 Materials and procedure

Students were contacted via email from the university’s research office and invited to participate in the survey. The survey was administered online on the university’s server using Microsoft Forms. Students filled in demographic data and completed the AGQ-R (Elliot and McGregor, 2001) and the StRIP (DeMonbrun et al., 2017). Students spent about 10 min answering the questions and were not compensated for participation.

### 3.3 Data analysis

Data were analyzed using repeated measures MANOVAs and calculating correlation coefficients using SPSS (IBM, Version 28). Differences between groups were assessed with repeated measures MANOVAs using SPSS (IBM, Version 28). Correlation coefficients for the relationships between motivation, engagement, and students’ perception were explored by calculating correlation coefficients, visually inspecting those and interpreting them given the evidence from previous studies described above.

### 3.4 Research ethics

This study was approved by the University’s IRB (264182). The research team followed established ethical guidelines and obtained online consent from participants. Ethical considerations were upheld in data collection, analysis, and reporting to ensure the trustworthiness of the study’s findings.

## 4 Results

### 4.1 Pre-service teachers’ motivational orientation

Since both the AGQ (Elliot and McGregor, 2001) and the StRIP (DeMonbrun et al., 2017) have 5 point Likert scales, but different numbers of questions per dimension, values reported here are normed to the 1 to 5 scale; higher numbers mean higher expression.

In total, students reported high levels of motivation (*M* = 3.9, *SD* = 0.83) with majority of 96 of students indicating high levels of motivation (averaged between 3.5 and 5), some (*N* = 34) reporting medium levels (averages between 2.5 and 3.49) and only few (*N* = 7) reporting low levels of motivation (averages below 2.49). Regarding the different versions of motivational orientation, only one student responded with minimum numbers for all 4 versions of motivation, and 25 students responded with maximum numbers for all 4 versions. The remaining 111 are in between and might show a preference for one over the other.

Participants in general indicated higher approach orientation (*M* = 4.1, SD = 0.94) than avoidance orientation (*M* = 3.7, SD = 1.13, *F_1, 136_* = 40.29, *p* < 0.001, *η^2^* = 0.229), but about the same levels of achievement (*M* = 3.9, SD = 1.10) and performance goal (*M* = 3.9, SD = 1.01, *F_1, 136_* = 1.08, ns, *η^2^* = 0.008). The interaction between both was statistically significant (*F_1, 136_* = 31. 48, *p* < 0.001, *η^2^* = 0.19), indicating that the difference between achievement approach orientation (*M* = 4.2, SD = 0.95) and achievement avoidance orientation (*M* = 3.5, SD = 1.14) is greater than the difference between performance approach (*M* = 4.0, SD = 0.93) and performance avoidance (*M* = 3.9, SD = 1.09) for the pre-service teachers. This means the majority of pre-teacher students are highly motivated, approach oriented, and pursue both achievement and performance goals.

### 4.2 Pre-service teachers’ perception of active instruction

Surprisingly, participants in this study reported significantly more passive instruction (*M* = 4.1, SD = 0.81) than all three versions of active instruction: constructive (*M* = 3.7, SD = 0.77), active (*M* = 3.7, SD = 0.82) and interactive instruction (*M* = 3.4, SD = 0.98; *F_3, 134_* = 25.81, *p* < 0.001, *η^2^* = 0.37).

### 4.3 Relationship between PSLTs’ perception of active instruction, motivation and engagement

All values indicating students’ engagement are on average higher than medium (*M_value_* = 3.7, SD*_value_* = 1.00; *M_positivity_* = 3.7, SD*_positivety_* = 1.01; *M_participation_* = 4.3, SD*_participation_* = 0.91), except for distraction (*M_distraction_* = 2.5, SD*_distraction_* = 1.18; *F*_3, 408_ = 94.08, *p* < 0.001, *η*^2^ = 0.41). Analysis of contrasts reveals that all positive aspects of engagement are statistically significantly different from distraction (*η*^2^ = 0.38 for value, *η*^2^ = 0.37 for positivity, and *η*^2^ = 0.54 for participation).

As this study is exploratory in nature, the reported significance levels should be interpreted as indicators of magnitude and strength of association only and not in the full sense of statistical inference testing.

In contrast, all positive aspects of engagement are positively correlated with one another, but not with distraction: *value* is correlated with *positivity* (*r* = 0.81, *p* < 0.001) and with *participation* (*r* = 0.61, *p* < 0.001), but not with distraction (*r* = 0.02, *ns*). Similarly, *positivity* is correlated with *participation* (*r* = 0.53, *p* < 0.001), but not with *distraction* (*r* = 0.02, *ns*). *Participation* is slightly negatively correlated with *distraction* (*r* = −0.16.02, *p* = 0.06).

Similarly, versions of *active instruction* are all highly positively correlated with one another: Perception of *active* instruction is correlated with *interactive* instruction (*r* = 0.75, *p* < 0.001), but also with *constructive* instruction (*r* = 0.76, *p* < 0.001) and even with *passive* instruction (*r* = 0.71, *p* < 0.001). Similarly, the perception of *interactive* instruction is correlated with *constructive* instruction (*r* = 0.64, *p* < 0.001) and with passive instruction (*r* = 0.50, *p* < 0.001). Finally, the perception of *constructive* instruction is correlated with *passive* instruction as well (*r* = 0.67, *p* < 0.001).

Eventually, all versions of *academic motivation* are positively correlated with one another as well: *mastery approach* orientation is correlated with *mastery avoidance* orientation (*r* = 0.46, *p* < 0.001), with *performance approach* orientation (*r* = 0.65, *p* < 0.001) and with *performance-avoidance* orientation (*r* = 0.54, *p* < 0.001). *Mastery avoidance* orientation is also correlated with *performance approach* orientation (*r* = 0.36, *p* < 0.001) and with *performance-avoidance* orientation (*r* = 0.53, *p* < 0.001). And finally, performance approach orientation is also correlated with performance-avoidance orientation (*r* = 0.83, *p* < 0.001).

Finally, the correlations between perceived versions of instruction, motivational orientation and engagement for identifying patterns of which version of instruction is appreciated by what kind of pre-service teacher resulting in different levels of engagement were inspected.

As expected, there are moderate to strong correlations between overall *motivation* and perception of *active instruction* (*r* = 0.44, *p* < 0.01), and between *motivation* and *engagement* (*r* = 0.38, *p* < 0.001), but not between perception of *active instruction and engagement* (*r* = 0.25, *p* < 0.01).

#### 4.3.1 Active instruction and motivation

**Active instruction** is positively correlated with all versions of motivation: *Mastery approach* (*r* = 0.47, *p* < 0.001), *mastery avoidance* (*r* = 0.34, *p* < 0.001), *performance approach* orientation (*r* = 0.34, *p* < 0.001), and *performance-avoidance* orientation (*r* = 0.29, *p* < 0.01). The rank order of correlation size is also expected, with *mastery orientation* being higher correlated with *active instruction* than *performance orientation* is.

In tendency, correlations between the perception of active instruction and versions of motivational orientation are bigger for *approach* than for *avoidance* orientation. The only surprise (see Table 1) is the very strong positive correlation between passive instruction and achievement approach orientation. This seems to indicate that students with achievement approach orientation actively listen to faculty’s instruction.

**Table 1:** Correlation matrix for versions of instruction (active, interactive, constructive; passive) and motivation (achievement goal orientation: *achievement approach* and *avoidance, performance approach* and *avoidance*).

Motivation/achievement goal orientation					
	Version	Achievement approach	Achievement avoidance	Performance approach	Performance avoidance
Instruction	Active	0.47**	0.34**	0.34**	0.29**
	Interactive	0.38**	0.34**	0.28**	0.19*
	Constructive	0.51**	0.36**	0.42**	0.36**
	Passive	0.60**	0.41**	0.34**	0.33**

Statistically highly significant correlations (*p* < 0.01) are marked with **, statistically significant correlations (*p* < 0.05) are marked with *.

##### 4.3.1.1 Active instruction and engagement

*Active instruction* is highly positively correlated with all versions of engagement: *value* (*r* = 0.53, *p* < 0.001), *positivity* (*r* = 0.54, *p* < 0.001), and *participation* (*r* = 0.45, *p* < 0.001). Engaged pre-service teachers perceive and highly appreciate active instruction. All correlations between perceived versions of instruction and aspects of engagement are positive and medium to strong. The only exception is the negative aspect of engagement, distraction. None of the versions is especially prone to distraction. Again, there is a surprising pattern for passive instruction that is similarly highly positively correlated with all positive aspects of engagement (see Table 2).

**Table 2:** Correlation matrix for versions of instruction (active, interactive, constructive; passive) and engagement (value, positivity, distraction).

Engagement					
	Version	Value	Positivity	Participation	Distraction
Instruction	Active	0.53**	0.54**	0.45**	0.06^ns^
	Interactive	0.38**	0.34**	0.30**	0.20*
	Constructive	0.45**	0.44**	0.46**	0.06^ns^
	Passive	0.64**	0.64**	0.57**	0.09^ns^

Statistically highly significant correlations (*p* < 0.01) are marked with **, statistically significant correlations (*p* < 0.05) are marked with *.

##### 4.3.1.2 Motivational orientation and engagement

The same holds for the correlations between engagement and versions of motivation: *Mastery approach* orientation (*r* = 0.30, *p* < 0.001), achievement avoidance orientation (*r* = 0.28, *p* < 0.001), *performance approach* orientation (*r* = 0.36, *p* < 0.001), and *performance avoidance* orientation (*r* = 0.32, 367, *p* < 0.001) are all positively correlated with *engagement*. As described above, most students are highly motivated and are not either achievement or performance oriented. They are eager to learn, but also want to score high. Therefore, it is not surprising that all versions of motivation are related to all positive aspects of engagement. Again, distraction has a different pattern than the positive aspects of engagement. There is a medium strong, negative correlation between achievement approach orientation, all other correlations are not meaningful (see Table 3).

**Table 3:** Correlation matrix for versions of motivation (achievement goal orientation: achievement approach and avoidance, performance approach and avoidance) and engagement (value, positivity, distraction).

Engagement					
	Version	Value	Positivity	Participation	Distraction
Motivation	Achievement approach	0.60**	0.49**	0.72**	−0.24**
	Achievement avoidance	0.36**	0.28**	0.34**	0.12^ns^
	Performance approach	0.40**	0.33**	0.51**	−0.01^ns^
	Performance avoidance	0.34**	0.28**	0.45**	0.04^ns^

Statistically highly significant correlations (*p* < 0.01) are marked with **, statistically significant correlations (*p* < 0.05) are marked with *.

To this end, it looks like, except for distraction, almost everything is correlated with everything: Motivation is correlated with engagement, but pre-service teachers are not either approach or avoidance oriented. In contrast, many students are both approach and avoidance oriented. For engagement, this makes even more sense: Who values active instruction, also feels positively toward instructor and class, actively participates, and is less distracted. That motivation, engagement, and active instruction are positively correlated with one another was expected as well.

## 5 Discussion

This study examined the students’ perception of active instruction and their motivational goals in tertiary-level pedagogy. It utilized descriptive survey research using the Achievement Goal Orientation – Revised (AGQ-R) and the Students Response of Instructional Practices (StRIP) surveys, and only correlations and group differences were tested.

The first research question focused on the motivational orientation of pre-service teachers in terms of achievement goal orientation. The majority of pre-service language teachers self-reported a high level of academic motivation, with a higher proportion reporting approach rather than avoidance orientation. The finding is consistent with the literature on approach orientation, which suggests that undergraduate students, pre-service and in-service teachers report being highly motivated in their academic and professional journey (Butler, 2007, 2012; Smith et al., 2013; Nguyen et al., 2021). While previous research predominantly found that pre-service and in-service teachers’ motivational goals are oriented toward mastery achievement (Castro-Villarreal et al., 2014; Wang et al., 2017; Nguyen et al., 2021), the results of the current study indicate that PSLTs in our sample have nearly equal levels of motivational goals, both mastery and performance goals.

In other words, pre-service language teachers in our study self-report tendencies to master the content and develop professionally, and at the same time, they expect praise and validation, good grades for completed assignments, and recognition from their instructors. The balance between these orientations highlights the complexity and diversity of motivational orientation influencing pre-service teachers in this educational context. Mastery and performance approach orientations are both manifestations of a desire to succeed in terms of achieving internally satisfying goals, producing a sense of achievement brought by knowledge acquisition, as well as by external measures of success, such as grades, praise, or other forms of recognition. The participants in the present study self-reported high mastery and performance approach orientations and significantly lower mastery and performance avoidance orientations in cross comparisons, which points to a very positive motivation for educational goal attainment. It is clear that further research is necessary to explore the contextual factors that contribute to this unique motivational profile.

The second research question focused on pre-service teachers’ perceptions of different types of classroom instruction. According to the study’s results, pre-service language teachers reported significantly more passive instruction than the three active instruction forms. The participants felt that the lecturing from the faculty was more passive, as indicated by a higher mean score and smaller standard deviation (*M* = 4.1, SD = 0.81). However, it is crucial to interpret these findings with caution due to two important reasons.

First, there is definitely room for passive instruction in higher education, especially in introductory courses as well as in the initial phase of advanced level courses when laying the necessary groundwork in preparation for more complex tasks requiring active student engagement (MacDonald and Frank, 2016). When the instructor is in control of the topic, content and the pace of delivery, providing passive training to learners may enhance their understanding of the active task ahead.

In addition, due to previous educational experience, students may be habituated to passively listen to lectures in school and at university. Therefore, they may interpret as “lecturing” any instantiation of faculty talking, including introduction, explanation, or guideline for active learning oriented tasks.

Second, the dividing line between active and passive instruction may be blurry. Especially in language education, the instructor’s verbal input is crucial even when implementing active instruction. The results of the study indicate a good balance or a healthy mix of the faculty talking to the students and students answering or performing in-class activities. It is too simplistic to categorize instruction as either active or passive, as teaching practices are more complex than that.

Regarding student engagement with active instruction, the findings indicate that the students were highly engaged in all areas, exhibiting positive cognitive and emotional traits and demonstrating high levels of participation. In addition, the results of the study indicate that students who are actively engaged in their studies tend to be less distracted during class.

This study aligned with prior research in the sense that students reported high engagement through active instruction, which may have helped them gain a deeper understanding of the content and develop their critical thinking skills (Freeman et al., 2014; Kuh et al., 2017; Owens et al., 2017).

The English Language Education program’s students receive the advantages of learning and practicing active instructions and are simultaneously equipped to become language teachers. This approach not only boosts their cognitive, educational, and emotional development as students but it also provides them with a blueprint for creating positive classroom experiences as future teachers (Wang et al., 2021). By embracing and applying all forms of active instruction, these future language educators can cultivate engaging and productive learning environments for their students.

The results indicate that for students with high achievement approach orientation, all the above reported correlations are true and strongly positive. They value active instruction, actively participate even in passive instruction, and they are much less prone to be distracted. For those students, active learning seems to be part of their identity. They strive for success and active instruction is their pathway to get where they want to be. For those students, constructive instruction is the most positive and they will even value and participate in less fun and more challenging activities. These students most likely even actively listen to passive instruction.

The one version that sticks out is the performance approach orientation: Students with high values do not necessarily report appreciation of active instruction, but they do report participation, especially if it is graded. According to self-determination theory (Deci and Ryan, 1985), the achievement may feel less rewarding. Students with such engagement profiles may not be interested in mastering the content through active instruction, but rather scoring high on exams, and they might potentially struggle with transferring knowledge from in-class activities to the exam. Responding to active instruction is a skill that needs to be learned as well. Maintaining academic motivation and grit in the face of challenges is difficult, especially with active instruction. The moment in-class activities are not graded, performance-oriented students might lose interest. They might not be excited about practicing new, unfamiliar skills and might not intuitively know what success in those situations looks like and feels like. In most values, students with high performance approach orientation look like students with high avoidance orientation. They might perform as well as achievement approach students in terms of grades, but they cannot harvest the full benefits that students with high achievement approach orientation get from active instruction, they do not enjoy the activities as much and they do not as easily construct knowledge and skills from those. Students with low motivation may find active instruction challenging because it is harder to estimate how to not fail the class. All that is easier with traditional, passive instruction because expectations and performance indicators there are explicit.

## 6 Conclusion

The current study has demonstrated that the pre-service language teachers in our sample report a higher approach goal orientation than avoidance goal orientation. In other words, the future language teachers we tested are more motivated by a desire to succeed rather than the fear of failure. Moreover, the participants in our sample report being equally motivated by both the acquisition of knowledge and skills (achievement orientation) and the rewards for and recognition of their achievement vis a vis their peers (performance orientation). While it is important to evaluate one’s abilities in terms of self-perceived satisfactory performance as indicative of competence, it is also crucial to draw meaningful comparisons between one’s own capacity and that of others, so that both internal and external measures of success can be integrated. In terms of classroom practices, pre- service language teachers report having experienced all types of instruction (passive, active, interactive and constructive) in the language education program, with passive instruction being significantly higher than all three types of active instruction. Crucially, although the pre-service language teachers’ perception of instruction as passive is preponderant in our sample, it does not necessarily point to a higher use of traditional passive lecture and note taking practices utilized by the program instructors. What it might reveal, however, is the PSLTs’ interpretation of any type of explanation-focused instruction as passive. More importantly, regardless of the perceived instruction type, the participants’ motivation and engagement remain high, as they report high cognitive and emotional engagement, as well as very high participation and low distraction levels. This is not surprising given that high engagement and motivation in the educational context typically leads to enhanced participation and diminished distraction levels.

Regarding correlations, motivation, engagement and active instruction are all highly correlated with each other, as highly motivated and engaged individuals tend to benefit more from active instruction than less motivated students, who chiefly rely on passive instruction for academic success.

### 6.1 Implications for pedagogy

Given the equally favorable perception of active and passive instruction with similar levels of motivation and engagement regardless of instruction type reported by the participants in our sample, it seems there is room for various pedagogical approaches in the modern language education classroom. The sampled PSLTs generally positively perceived both active and passive strategies utilized in the course of their language education program, which may indicate an emergent need for a careful balance between various teaching strategies and approaches in language education programs in particular, and possibly at tertiary level pedagogy in general. As students’ motivation and engagement levels necessarily vary within and across university programs, balancing passive and active instruction and varying educational approaches might ensure better knowledge and skills acquisition, as well as laying the foundations for future academic success. Less motivated and engaged students who initially experience reluctance toward active learning strategies might in time gain confidence and independence by solely relying on passive instruction while carefully scaffolding the fundamentals of knowledge and skills necessary for advancement in the program.

Experiencing academic success in this way could be a necessary precursor to creating a favorable reception of active learning strategies at a later stage.

### 6.2 Limitations of the current study and directions for further research

The current study is part of a dataset involving responses from participants in various university majors and from all university types (state, private) in several GCC countries. The study reports on results obtained from a relatively small subset of the participants representing only one university major (English Language Education) and one university type (private with the American curriculum) in Kuwait. Therefore, the findings cannot be generalized to other majors or even to the general student population in Kuwait or other GCC countries.

The study uses combined quantitative measures (questionnaires) and explores novel multi-layered correlations between the measured variables. In addition to the quantitative approaches using closed-ended survey questions, which probe into self-reported perceptions of pedagogical strategies and related variables, the research on active and passive instructions and their relationships to motivation, achievement goal orientation and engagement could benefit from an in-depth, qualitative approach with open-ended questions and more direct investigative techniques, such as observations and focus groups. Also, our sampling technique has allowed us to capture the extant engagement, motivation levels and correlations between them at one point in time. However, as engagement and motivation are dynamic constructs bound to vary throughout the duration of any academic program, it might be insightful to conduct a longitudinal study focusing on the instruction type at various stages of the program and the related measured of the variables under consideration.

## Data availability statement

The original contributions presented in the study are included in the article/supplementary material, further inquiries can be directed to the corresponding author.

## Ethics statement

The studies involving humans were approved by IRB of the Gulf University for Science and Technology in Kuwait. The studies were conducted in accordance with the local legislation and institutional requirements. The participants provided their written informed consent to participate in this study.

## Author contributions

FH: Conceptualization, Formal analysis, Funding acquisition, Investigation, Methodology, Project administration, Resources, Supervision, Visualization, Writing – original draft, Writing – review & editing. MT: Conceptualization, Formal analysis, Investigation, Project administration, Resources, Visualization, Writing – review & editing. AB: Conceptualization, Data curation, Formal analysis, Investigation, Methodology, Software, Validation, Visualization, Writing – review & editing.
